# Analyzing genetic diversity in luffa and developing a Fusarium wilt-susceptible linked SNP marker through a single plant genome-wide association (sp-GWAS) study

**DOI:** 10.1186/s12870-024-05022-7

**Published:** 2024-04-22

**Authors:** Yun-Da Li, Yu-Chi Liu, Yu-Xuan Jiang, Ahmed Namisy, Wen-Hsin Chung, Ying-Hsuan Sun, Shu-Yun Chen

**Affiliations:** 1 Department of Agronomy, National Chung-Hsing University, Taichung, Taiwan; 2 Department of Plant Pathology, National Chung-Hsing University, Taichung, Taiwan; 3 Department of Forestry, National Chung-Hsing University, Taichung, Taiwan

**Keywords:** Single-plant GWAS, Luffa, Fusarium wilt, ddRAD, Susceptible, SNP marker

## Abstract

**Background:**

Luffa (*Luffa* spp.) is an economically important crop of the Cucurbitaceae family, commonly known as sponge gourd or vegetable gourd. It is an annual cross-pollinated crop primarily found in the subtropical and tropical regions of Asia, Australia, Africa, and the Americas. Luffa serves not only as a vegetable but also exhibits medicinal properties, including anti-inflammatory, antidiabetic, and anticancer effects. Moreover, the fiber derived from luffa finds extensive applications in various fields such as biotechnology and construction. However, luffa Fusarium wilt poses a severe threat to its production, and existing control methods have proven ineffective in terms of cost-effectiveness and environmental considerations. Therefore, there is an urgent need to develop luffa varieties resistant to Fusarium wilt. Single-plant GWAS (sp-GWAS) has been demonstrated as a promising tool for the rapid and efficient identification of quantitative trait loci (QTLs) associated with target traits, as well as closely linked molecular markers.

**Results:**

In this study, a collection of 97 individuals from 73 luffa accessions including two major luffa species underwent single-plant GWAS to investigate luffa Fusarium wilt resistance. Utilizing the double digest restriction site associated DNA (ddRAD) method, a total of 8,919 high-quality single nucleotide polymorphisms (SNPs) were identified. The analysis revealed the potential for Fusarium wilt resistance in accessions from both luffa species. There are 6 QTLs identified from 3 traits, including the area under the disease progress curve (AUDPC), a putative disease-resistant QTL, was identified on the second chromosome of luffa. Within the region of linkage disequilibrium, a candidate gene homologous to *LOC111009722*, which encodes peroxidase 40 and is associated with disease resistance in *Cucumis melo*, was identified. Furthermore, to validate the applicability of the marker associated with resistance from sp-GWAS, an additional set of 21 individual luffa plants were tested, exhibiting 93.75% accuracy in detecting susceptible of luffa species *L. aegyptiaca* Mill.

**Conclusion:**

In summary, these findings give a hint of genome position that may contribute to luffa wild resistance to Fusarium and can be utilized in the future luffa wilt resistant breeding programs aimed at developing wilt-resistant varieties by using the susceptible-linked SNP marker.

**Supplementary Information:**

The online version contains supplementary material available at 10.1186/s12870-024-05022-7.

## Introduction

Luffa (*Luffa* spp.), also known as sponge gourd or vegetable gourd, is an annual cross-pollinated economic crop belonging to the Luffa genus within the Cucurbitaceae family [1]. Within this genus, there are five to eight species; however, only *L. acutangula Roxb*. and *L. aegyptiaca Mill*. have been domesticated for different applications, and their genome information has been revealed. Both of these species possess diploid genomes with 13 pairs of chromosomes (2*n* = 26), encoding approximately 25,508 proteins. The genome size is 734.6 Mb for *L. acutangular* and 656.19 Mb for *L. aegyptiaca* [2, 3].

Luffa has found wide-ranging applications, not only for food consumption with fresh fruit, but also for its dried fruit and fiber, which offer attributes like high strength, lightweight, low cost and sustainable utilization. These properties make them excellent materials for various fields including biotechnology [4], construction [5] and environmental engineering [6]. Additionally, the extracts derived from Luffa have demonstrated medicinal potential in addressing diabetes [7], inhibit inflammation [8], treating cancer [9], combating bacteria [10], and fighting fungi [11]. Overall, Luffa shows great potential to improve various aspects of our lives [12]. However, luffa production faces substantial losses due to luffa Fusarium wilt, a severe disease caused by the fungus *Fusarium oxysporum* (Fo). This resilient fungus can persist in the soil for an extended period, and infected plants display symptoms such as slow growth, yellowing of lower leaf, and browning of vascular bundles, ultimately leading to reduced yield and plant fatalities [13]. While several control methods are currently available, many of them are unsuitable due to cost, effectiveness, and environmental concerns. Therefore, there is an urgent imperative to breed Fusarium wilt-resistant luffa varieties for future commercial luffa production.

Genome-wide association study (GWAS) stands as a powerful and efficient tool for unraveling the intricate relationships between diverse genotypes and phenotypes. It allows for the discovery of quantitative trait loci (QTLs) and the identification of candidate genes that influence phenotypic traits. Besides, GWAS aids in the development of trait-linked markers for marker assisted selection (MAS) during the breeding process [14]. GWAS has successfully unveiled numerous disease-resistant QTLs in various crops. For instance, in maize, 18 novel head smut resistance candidate genes were identified [15], while in barley, 9 QTLs related to spot blotch resistance were revealed [16]. In soybean, a total of 27 QTLs associated with resistance to white mold were discovered [17]. Moreover, GWAS has been applied in wheat to discover novel QTLs resistant to stripe rust [18]. Furthermore, GWAS has proven to be a valuable tool in uncovering QTLs and candidate genes for Fusarium resistance in crops such as wheat [19–21], maize [22–24], rye [25], barley [26], oat [27], radishes [28], strawberry [29], and cotton [30, 31] in recent studies. However, GWAS typically requires a panel of inbred lines with multiple individuals for phenotypic analysis in crops, which necessitates time for population development. Single-plant GWAS (sp-GWAS) has been successfully applied in maize [32], wheat [33], lentil [34], and pea [35], demonstrating its ability to identify candidate genes associated with phenotype variation by performing GWAS on individual plants. This approach reduces the time and resources required to complete the GWAS [32].

In this study, we analyzed the phenotypes of 97 individuals representing 73 luffa accessions encompassing two major luffa species, *L. acutangula* (L) Roxb. and *L. aegyptiaca* Mill, in order to conduct a sp-GWAS aimed at identifying markers and candidate genes associated with three traits, including the Fusarium wilt-resistance trait. Our investigation led to the discovery of a QTL on chromosome 2, containing a candidate gene that may encode a homologue of peroxidase 40, which plays a role as disease resistance gene in *Cucumis melo*. Furthermore, we developed an associated SNP marker and validated its accuracy in detecting susceptible luffa plants.

## Results

### Distribution patterns of material resources, phenotypes, and correlation analysis among 73 luffa accessions

A total of 97 individuals were gathered from 73 luffa accessions. Approximately 65% of the materials were attributed to *L. aegyptiaca* Mill, while 34% belonged to *L. acutangula* (L) Roxb. These luffa materials were sourced from 11 countries, including the Philippines (34 individuals, 35%), Laos (12 individuals, 12%), Thailand (12 individuals, 12%), Taiwan (10 individuals, 10%), Vietnam (10 individuals, 10%), Malaysia (9 individuals, 9%), Indonesia (4 individuals, 4%), Bangladesh (3 individuals, 3%). Furthermore, only 1 individual each was collected from Cambodia, Uzbekistan and USA (1% of each) (Table S1).

**Fig. 1:**
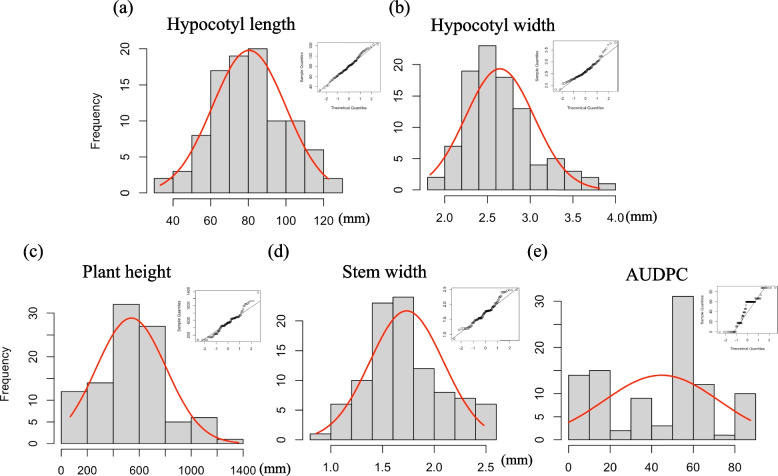
Diverse phenotype distributions were observed among the 97 luffa individuals. The distribution of distinct phenotypes was recorded for various traits including (**a**) hypocotyl length; (**b**) hypocotyl width; (**c**) plant height; (**d**) stem width; and (**e**) AUDPC. The smooth density curve is represented by the red line, while the qqplot located in the upper right corner of each sub-figure illustrates the univariate normality of specific phenotypes

In addition, this research documented a total of 5 distinct quantitative phenotypes and one qualitative trait across the 73 luffa accessions. The distribution and qqplot for the normal distribution test of quantitative phenotypes were presented in Fig. 1. Among these phenotypes, only hypocotyl length closely approximated a normal distribution, while the other four traits exhibited imperfect fits.

**Fig. 2:**
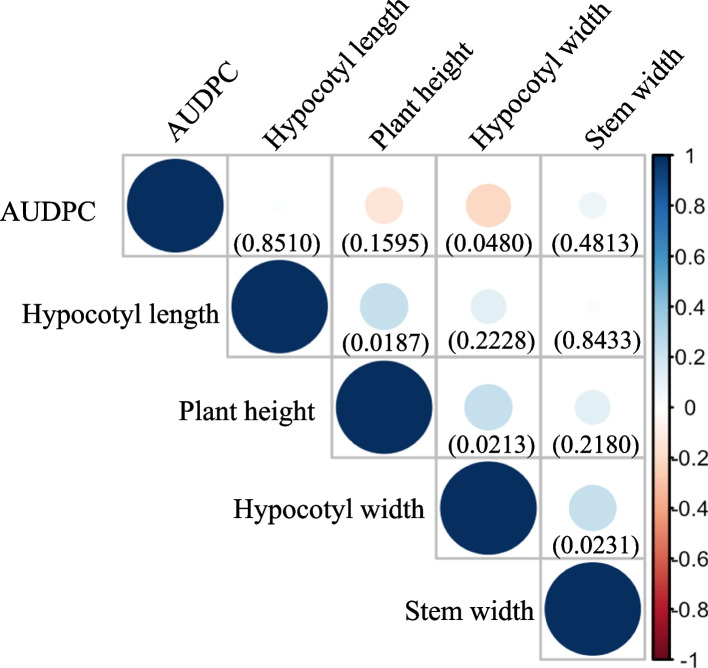
The correlation among all recorded phenotypes. The correlation among all phenotypes was estimated individually, displaying the R^2^ value in different color. Red indicates a negative correlation, while bule represents a positive correlation. The *p*-value for each relationship is indicated by a number at the button of each correlation square

Correlation analysis was carried out among all traits and is illustrated in Fig. 2. The results revealed significant but relatively low correlations. A negative correlation was observed between the trait AUDPC and hypocotyl width (R^2^ = -0.2, *p*-value = 0.048), while positive correlations were observed between hypocotyl length and plant height (R^2^ = 0.24, *p*-value = 0.0187), plant height and hypocotyl width (R^2^ = 0.23, *p*-value = 0.0213), as well as hypocotyl width and stem width (R^2^ = 0.23, *p*-value = 0.0231).

### PCA analysis and population structure detection using STRUCTURE after SNP calling

**Fig. 3:**
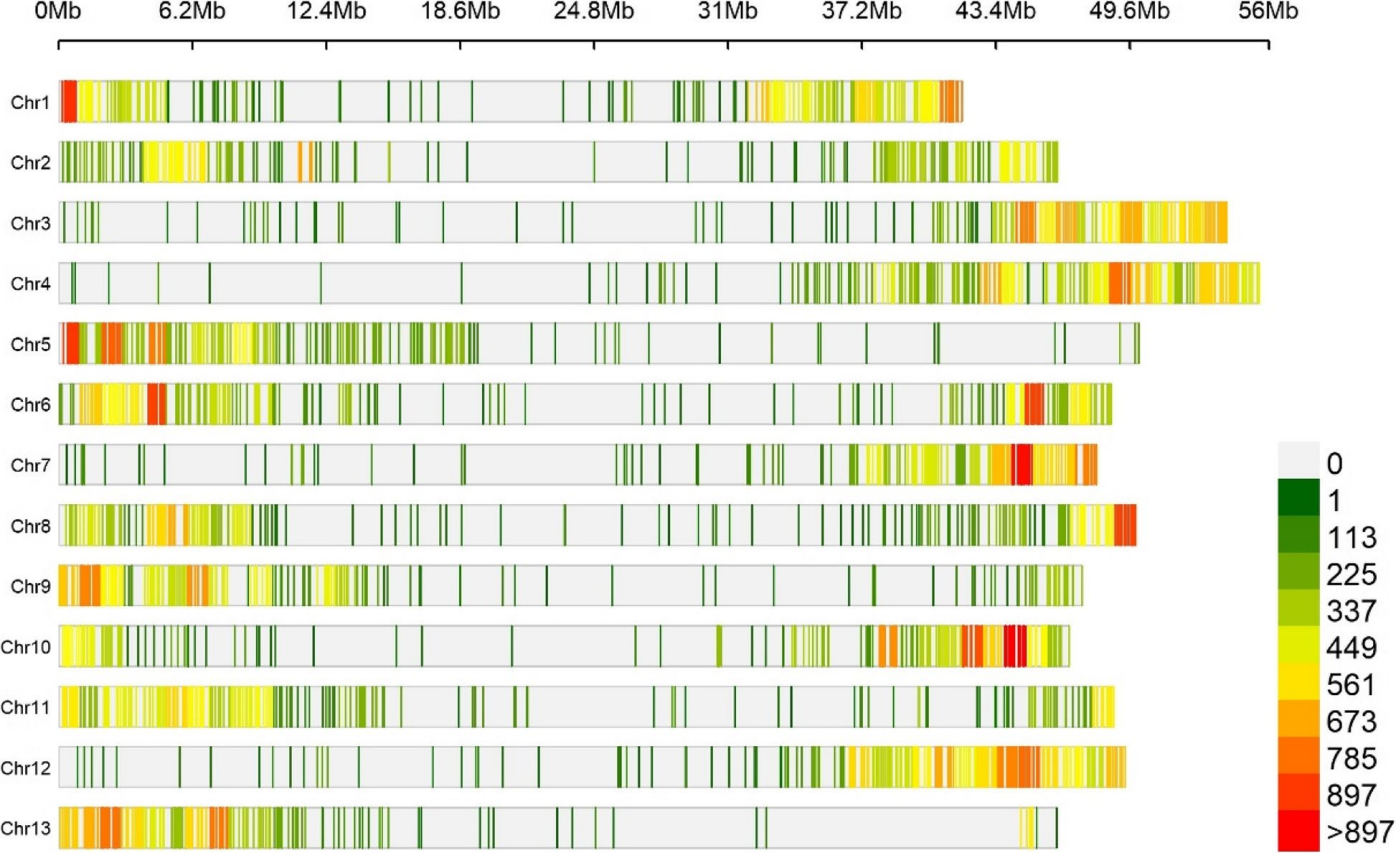
Distribution of high-quality SNPs applied in this study. The distribution of 8,819 high-quality SNPs across 13 chromosomes of luffa is illustrated. The color represents the quantity of SNPs within specific locations on the chromosome

The acquisition of a total of 8,919 high-quality genome-wide SNPs were accomplished through ddRAD library sequencing, achieving a 12% breadth of coverage, and we displayed the distribution of SNPs was showed in Fig. 3. The distribution of SNPs on each chromosome showed that chromosome 4 contains the highest number of 766 SNPs, while chromosome 13 showed the least 610 SNPs. Besides, the average distance between one SNP was longest on chromosome 3 at 85.2 Kb and the shortest on chromosome 1 at 57.22 Kb, with an overall average of 75.09 Kb (Table 1).

**Table 1:** Single nucleotide polymorphism (SNP) marker distribution among luffa chromosomes

Chromosome	Length (Mb)	Numbers of SNPs	Average length (kb/SNP)
Chr1	42.17	737	57.22
Chr2	46.43	674	68.89
Chr3	54.10	635	85.20
Chr4	55.64	766	72.64
Chr5	50.54	652	77.52
Chr6	48.76	743	65.63
Chr7	48.28	631	76.51
Chr8	50.08	664	75.42
Chr9	47.39	647	73.25
Chr10	46.82	719	65.12
Chr11	48.96	692	70.75
Chr12	49.62	749	66.25
Chr13	47.31	610	77.56
Total	669.70	8,919	75.09

**Fig. 4:**
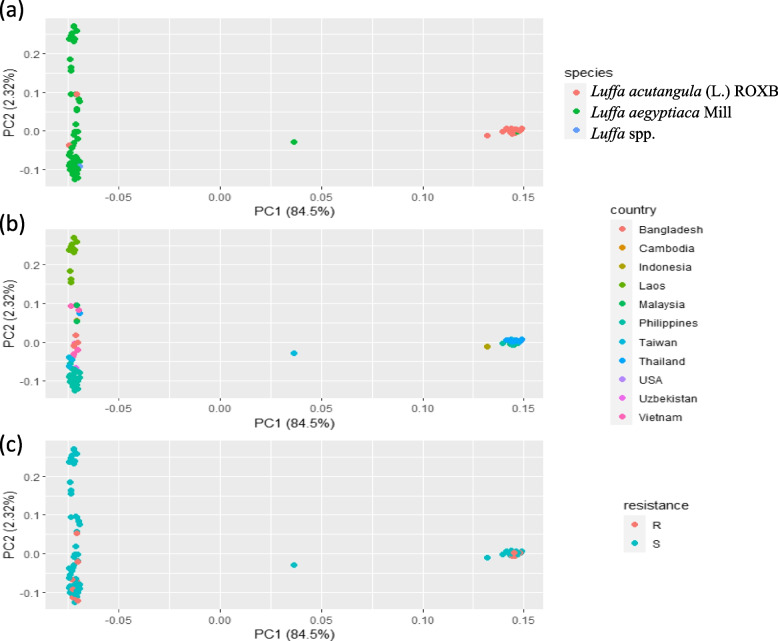
The PCA analysis among 97 luffa individuals based on high-quality SNPs. PCA analysis was conducted on the group of 97 luffa individuals. To delve into the specifics of these accessions, the analysis was further explored with respect to (**a**) luffa species; (**b**) source countries; and (**c**) resistance to *Fusarium oxysporum*

The subsequent PCA analysis was executed using the complete genotypic dataset comprising 97 luffa individuals, and the results are visualized in Fig. 4. To achieve a comprehensive understanding of these accessions, the analysis was further explored in relation to luffa species (Fig. 4A), source countries (Fig. 4B) and resistance to *Fusarium oxysporum* (Fig. 4C) after the PCA analysis. The PCA analysis revealed three distinct groups, which predominantly corresponded to their respective luffa species (Fig. 4A). It’s worth noting that the species *L. acutangular* (L.) ROXB was predominantly associated with accessions from Taiwan (Fig. 4B). Moreover, a pivotal discovery was made, indicating that accessions belonging to both *L. acutangular* (L.) ROXB. and *L. aegyptiaca* Mill demonstrated resistance to *Fusarium* after inoculation (Fig. 4C). For a more detail perspective, among the individuals of *L. aegyptiaca* Mill, 9 (14.3%) demonstrated resistance to Fusarium wilt 21 days after inoculation, while in *L. acutangula* (L.) ROXB., 5 (15.2%) samples exhibited resistance. Additionally, out of the 9 luffa materials from Malaysia, 4 exhibited the resistance to Fusarium wilt, representing the highest ratio (44.4%) of resistant individuals, whereas samples from Laos did not display any resistance among the 12 individuals (0%) (Table S1).

**Fig. 5:**
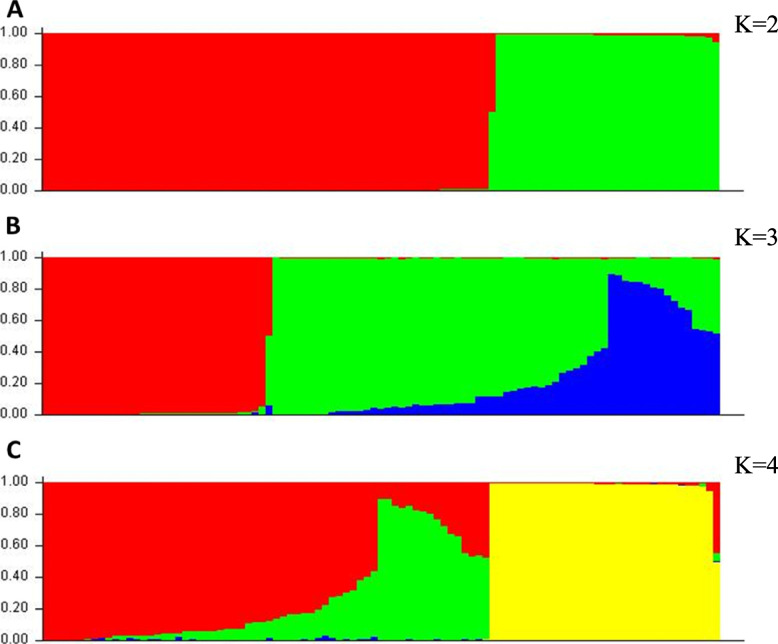
Population structure obtained by Structure software from 97 luffa individuals for different values of clusters (K). Population structure was conducted among 97 luffa individuals including 73 accessions by structure software with distinct clusters: (**a**) K = 2; (**b**) K = 3; and (**c**) K = 4. Each individual is represented by a vertical bar

These findings suggest that the collected luffa accessions exhibit significant genetic diversity, with a specific focus on the trait of resistance to Fusarium invasion. Furthermore, the population structure estimation encompassed 97 individuals, revealing a robust population stratification, as illustrated in Fig. 5, and supporting the choice of K = 2 as the basis for selection.

### GWAS results and identifying candidate genes

**Fig. 6:**
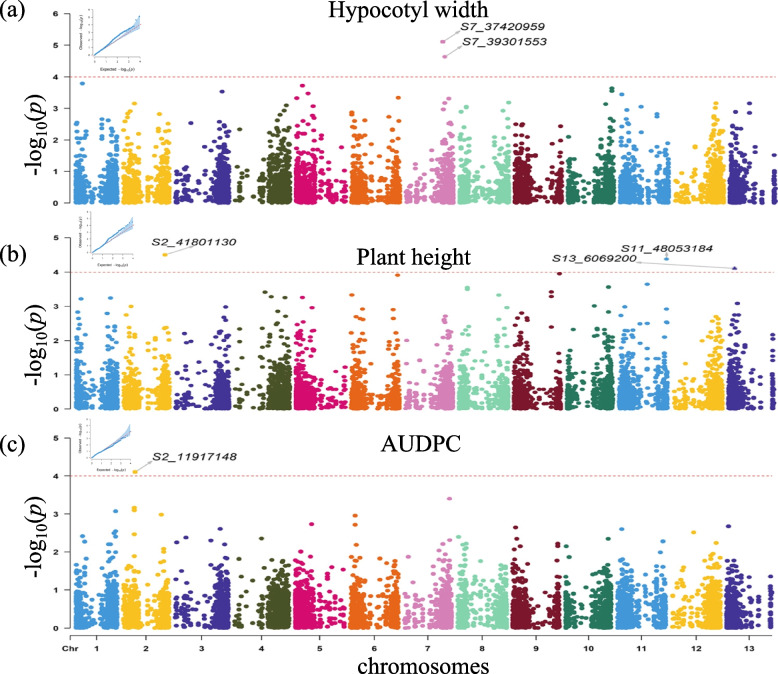
The Manhattan plot of three luffa traits. Following the sp-GWAS analysis, a Manhattan plot was conducted for various luffa traits: (**a**) Hypocotyl width; (**b**) plant height; (**c**) AUDPC. In each sub-figure, the significant trait-associated SNPs were depicted, and a qq-plot of each analysis was presented in the upper left corner

**Fig. 7:**
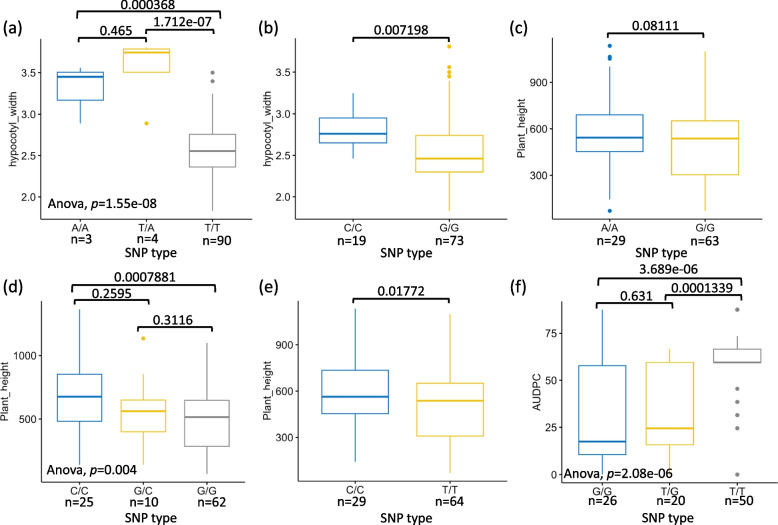
Comparing recorded phenotypes based on different SNP types within trait-associated SNPs. To comprehend the distinctions attributed to different SNP types within trait-associated SNPs identified after sp-GWAS, the phenotypes of various traits were segregated. This separation aimed to uncover the impact of distinct trait-associated SNP types. Specifically: (**a**) hypocotyl width associated SNP S7_37420959; (**b**) hypocotyl width associated SNP S7_39301553; (**c**) plant height associated SNP S2_41801130; (**d**) plant height associated SNP S11_48053184; (**e**) plant height associated SNP S13_6069200; (**f**) AUDPC associated SNP S2_11917148. The *p*-value of each comparison is provided above the brackets, while the number of individuals associated with each SNP is indicated under different SNP types

Five different traits, including AUDPC, hypocotyl length, hypocotyl width, plant height, and stem width, were utilized as phenotype data in GWAS, employing a general linear model (GLM) augmented by the incorporation of five PCAs to account for population structure within the collected luffa individuals. The correction level was set at *p*-value = 0.0001 as the criteria for identifying trait-associated QTLs. Notably, six QTLs potentially contributing to hypocotyl width (2 QTLs), plant height (3 QTLs), and AUDPC (1 QTL) were revealed and illustrated in Fig. 6. It’s evident that most of these QTLs exhibit discernible differences in the distribution of phenotypes according to SNP types, as illustrated in Fig. 7a, b, d to f. Nonetheless, it is noteworthy that one SNP (S2_41801130) associated with plant height did not exhibit such differentiation, as depicted in Fig. 7c.

The identification of candidate genes within each QTL was pursued within a genomic region of 10 K upstream and downstream of the trait-associated SNPs. Putative genes with annotated functions were listed and summarized in Table 2. Firstly, genes with homology to *LOC127143780* and *LOC111469808*, which encode a SNF-1 related protein kinase regulatory subunit gamma-1-like and an F-box protein, respectively, were linked to the regulation of hypocotyl width. Additionally, genes presumed to influence plant height were discovered, with homologous to *LOC111459237*, *LOC111809662*, and *LOC111009991*, encoding monocopper oxidase-like protein SKU5, cold shock domain-containing protein 3-like, and U-box domain-containing protein 44-like, respectively. Furthermore, the gene homologous to *LOC111009722*, which encodes peroxidase 40, exhibited a notable association with the AUDPC trait.

**Table 2:** The trait-associated SNPs and candidate genes for different phenotypes based on GWAS analysis

Trait	SNP ID	Chromosome and position	p-value	R^2^(%)	Candidate gene	Functional annotation
Hypocotyl width	S7_37420959	7:37420959	7.79E-06	12.68	*LOC127143780*	SNF1-related protein kinase regulatory subunit gamma-1-like
	S7_39301553	7:39301553	2.33E-05	11.52	*LOC111469808*	F-box protein
Plant Height	S2_41801130	2:41801130	3.16E-05	18.48	*LOC111459237*	Monocopper oxidase-like protein SKU5
	S11_48053184	11:48053184	4.19E-05	17.83	*LOC111809662*	Cold shock domain-containing protein 3 -like
	S13_6069200	13:6069200	7.91E-05	16.78	*LOC111009991*	U-box domain-containing protein 44-like
AUDPC	S2_11917148	2:11917148	7.83E-05	15.40	*LOC111009722*	Peroxidase 40

### Validation of AUDPC-associated SNP marker

A SNP marker potentially associated with AUDPC was developed using the position of AUDPC-associated SNP (Table S2). To determine its effectiveness in discerning susceptibility or resistance to Fusarium wilt in luffa plants, a total of 21 *L. aegyptiaca* Mill individuals were employed for the validation program. Phenotype assessment occurred 21 days after inoculation, with phenotype classification based on disease rank: rank 0–1 were classified as resistant, and ranks exceeding 2 were classified as susceptible. Among the 21 individuals, 16 samples exhibited a susceptible phenotype, while 5 individuals displayed a resistant phenotype after *Fusarium* inoculation. When subjecting the specimens to PCR for SNP typing, an accuracy of approximately 76.19% was observed when considering both susceptible and resistant phenotypes. Notably, a high accuracy (93.75) was evident in predicting susceptibility compared to resistance (Table 3).

**Table 3:** The accuracy of AUDPC-associated marker evaluated among 21 individuals of *Luffa aegyptiaca* Mill

Phenotype	Number of individuals	Number of SNP matched with phenotype	Number of SNP not matched with phenotype	Accuracy (%)
Susceptible	16	15	1	93.75
Resistant	5	1	4	20.00
Total	21	16	5	76.19

## Discussion

### Enhanced resistance to Fusarium wilt in robust luffa plants

The hypocotyl, as the subsequent organ emerging from the seed, facilitates increased exposure of the seedling to sunlight for photosynthesis [36]. In our study, we identified three notable yet moderately positive correlations: between hypocotyl length and plant height, between hypocotyl width and plant height, and between hypocotyl width and stem width. These findings imply that a more robust hypocotyl may result in a healthier plant. This observation aligns with the concept that hypocotyl attributes, including both length and width, play a crucial role in seedling emergence [36]. Moreover, a negative correlation was observed between AUDPC and hypocotyl width, emphasizing that a sturdy luffa plant could mitigate the impact of *Fusarium* invasion.

### Variation in Fusarium wilt resistance across different countries in both luffa species

Resistance to Fusarium wilt was identified in both luffa species examined in this study. Specifically, a resistance rate of 14.3% was observed in *L. aegyptiaca* Mill, while *L. acutangular* (L.) ROXB. demonstrated a resistance rate of 15.2%. This discovery suggests that individuals displaying resistance within each species could potentially serve as valuable genetic material for future luffa Fusarium-resistant breeding programs.

It’s essential to emphasize that luffa samples from Malaysia exhibited the highest ratio of plants resistant to Fusarium wilt, while none of the sample from Laos showed resistance. Beyond the potential influence of our sample collection methods and the specific *Fusarium* species or isolates used for inoculation, which may impact luffa resistance [37], another plausible explanation for this difference could be the influence of the plants’ geographic origins. The country where luffa plants originated might significantly affect their ability to resist Fusarium wilt due to geographical isolation. This finding aligns with previous research indicating significant variations in luffa’s resistance to Fusarium wilt across different countries. For instance, there were no instances of Fusarium wilt-resistant luffa in Cambodia and Uzbekistan [38].

All these findings suggest that inclusion of hypocotyl traits and consideration of the geographical origin of the material could be promising avenues for selecting better breeding material in future luffa Fusarium-resistant breeding programs.

### Candidate genes within QTLs for different traits in Luffa

In the current study, a total of 6 QTLs were identified across 3 distinct traits, specifically hypocotyl width, plant height, and AUDPC. Initially, two homologous genes, namely *LOC127143780* and *LOC111469808*, were detected in relation to the hypocotyl width-associated SNPs S7_37420959 and S7_39301553. The former gene encodes a SNF1-related protein kinase regulatory subunit gamma1-like, which plays a role in carbohydrate metabolism and ABA signal transduction in plants [39]. The latter homologous gene is responsible for encoding F-box proteins, constituting one of the largest protein families discovered in plants, with a diverse range of functions and broad coverage. F-box proteins are integral to various plant responses, including stress adaptation, development, regulation of plant hormones, and the biosynthesis of secondary metabolites [40]. Additionally, F-box proteins have been proven to correlate with hypocotyl elongation in *Arabidopsis* as well [41].

Three QTLs associated with plant height were identified, positioned on chromosome 2, 11, and 13, corresponding to SNP S2_41801130, S11_48053184, and S13_6069200, respectively. These SNPs were used to identify the associated candidate genes, which were determined as *LOC111459237, LOC111809662,* and *LOC111009991* based on sequence homology analysis. The *LOC111459237* gene encodes the monocopper oxidate-like protein SKU5, actively involved in oxidative-reductive reactions within organisms, Notably, *SKU5* gene also plays a role in facilitating cell wall elongation [42, 43]. Additionally, intriguing negative correlations between *SKU5* expression and both plant height and leaf area were discovered in alfalfa (*Medicago sativa* L.) [44]. The *LOC111809662* gene is responsible for encoding the cold shock domain-containing protein 3-like, which plays a role in plant tolerance to low-temperature stress. Furthermore, it is abundantly expressed in meristematic and developmental tissues, indicating its potential involvement in plant growth and development [45]. Lastly, the final candidate gene, *LOC111009991,* encodes a protein housing the U-box domain, which is involved in ubiquitination processes within plants and may play an important role in the plant disease resistance network. Notably, proteins featuring the U-box domain are responsible for the degradation of misfolded proteins [46]. Moreover, the role of U-box domain-containing proteins extends to the regulation of cell proliferation and division in the *Arabidopsis* root meristem [47], as well as being linked to processes like cell elongation, which exert influences on diverse phenotypes, including plant height in rice [48].

The candidate gene within the AUDPC-associated QTL region is *LOC111009722*, which encodes peroxidase, an enzyme known for its metabolic activity against reactive oxygen species (ROS). This enzymatic role enhances plant tolerance to both biotic and abiotic stresses. Additionally, peroxidase actively participates in various physiological responses within the plant [49]. Evidence substantiate that genes encoding peroxidase play a crucial role in plant’s response to pathogen. For instance, distinct expression levels of various peroxidase isozymes can influence resistance to downy mildew disease in pearl millet [50]. Furthermore, disparities in peroxidase synthesis timing between Malvaceae species exhibiting resistance and susceptibility have been correlated with distinct responses to the pathogen *Verticillium dahlia* [51]. Moreover, the overexpression of rice cationic peroxidase in carrots has led to heightened resistance against necrotrophic foliar pathogen [52]. These examples collectively suggest that the candidate genes identified may contribute to the specific phenotype under consideration. Nevertheless, further analysis, including complete gene sequencing and qRT-PCR of candidate genes, is indispensable to definitively ascertain the authentic gene function and to create a functional GMO in a model plant in the future.

### Validation of the AUDPC-associated SNP marker

Once QTLs are identified, the associated markers for specific traits are expected to be consistently applied for marker assisted selection (MAS) in the next step. Therefore, the accuracy of these linked markers in relation to the target trait becomes very important and necessitates assessment before their practical application. In this investigation, an AUPDC-linked SNP marker was developed and subsequently validated using a set of 21 individuals from the *L. aegyptiaca* Mill species. The results revealed a precision of 93.75% in detecting susceptible individuals while achieving an overall accuracy of 76.19%. However, the accuracy for identifying resistant individuals was only 20%. This outcome demonstrated robust discriminatory capability exclusively for a singular phenotype category. Similar findings have been observed in wheat, wherein linked markers to the *Lr19* gene showed polymorphism solely among susceptible cultivars [53]. Another illustrative example is the SNP marker linked to *HsBvm-1 in* sugar beet, which exclusively manifests in tolerant varieties and distinguishes among a singular phenotype [54]. Moreover, it is plausible that recombination events between the linkage marker and the authentic trait-controlling gene contribute to the observed lack of absolute accuracy in the present investigation [55].

## Conclusion

In this investigation, we have confirmed the presence of Fusarium wilt-resistant resources within two distinct Luffa species. Furthermore, we successfully identified a total of six QTLs associated with three distinct traits, and within the genomic regions of these QTLs, candidate genes were predicted. Notably, we developed and validated an SNP associated with AUDPC, indicating resistance to Fusarium wilt. These analyses displayed notably high accuracy in identifying susceptible individuals of the *L. aegyptiaca* Mill species. The acquisition of such valuable insights holds promising implications for the future cultivation of robust luffa cultivars that can effectively combat Fusarium wilt.

## Materials and methods

### Plant materials and leaf DNA extraction from Luffa accessions

In this study, we obtained two luffa species: *L. aegyptiaca* Mill and *L. acutangula* (L) Roxb., a total of 97 individuals, comprising 73 accessions, were acquired from the World Vegetable Center (93 individuals) and supported by KNOWN-YOU Seed Co. LTD. as a gift (4 individuals) (Table S1). Luffa plants were cultivated in the green house at NCHU (24°07′12.4"N, 120°40′32.9"E) in the year 2022. The DNA of each individual was extracted from young leaves using a modified cetyltrimethylammonium bromide (CTAB) method based on Doyle and Doyle (1987) [56].

### Evaluation of wilt symptoms after *Fusarium* inoculation and recording other phenotypes in luffa

A total of 97 individuals, including 73 accessions, were subjected to the evaluation of wilt symptoms, supported by Dr. Chung’s lab at NCHU. *Fusarium oxysporum* f. sp. *luffae*: (FOSULT) was applied approximately 4 weeks after germination, when 1–2 true leaves have developed. For inoculation, the luffa plant were removed from 2.5-inch growth pods and their roots were cleaned with water. Subsequently, the root were cut to retain only one-third of their original length. The cut roots were then soaked in a buffer containing Fusarium spore suspension (2.5 × 10^6^ spores/mL) for 30 min before being replanted in pots with clean soil mixture (peat: vermiculite = 3:1) for cultivation. Symptom evaluation was conducted 7, 14, and 21 days after inoculation, and disease severity was classified on a scale from 0 to 5 [57] (where level 0 indicates no symptom, level 1 represents yellowing or wilting of cotyledons; level 2 indicates yellowing or wilting of the first true leaf; level 3 indicates the yellowing or wilting of two true leaves; level 4 indicates yellowing or wilting of at least half of plant leaf; and level 5 indicates plant death). After recording disease severity at 21 days post- inoculation, the resistance status of each individual was determined based on their disease rank: individuals with ranks ranging from 0 to 1 were classified as resistant, while those with ranks exceeding 2 were classified as susceptible. In addition, the area under the disease progress curve (AUDPC) was estimated for each individual using the following formula, where *y*_*i*_ represents the severity of the disease level at the *i*-th observation, *x*_*i*_ corresponds to the day of the *i*-th observation, and *n* indicates the total count of observations [58].

$$AUDPC=\sum_{{\text{i}}=1}^{{\text{n}}}\left[\left(\frac{{{\text{y}}}_{{\text{i}}}+{{\text{y}}}_{\dot{{\text{i}}}-1}}{2}\right)\left({{\text{x}}}_{{\text{i}}}-{{\text{x}}}_{{\text{i}}-1}\right)\right]$$

There are four traits estimated other than the AUDPC on 21 days, which including hypocotyl length, hypocotyl width, plant height, and stem width. The “mice” package [59] was applied to address missing value of these four traits through multiple imputations within the R platform.

### Genotyping and SNP calling in luffa

In accordance to the experimental protocols outlined by Peterson et al. (2012) [60] and Shirasawa et al. (2016) [61] for ddRAD-seq, genomic DNA underwent digestion using two restriction enzymes, *Msp*I (NEB) and *Pst*I-HF (NEB). The resulting digested DNA was then ligated with adapters, subjected to size selection, and amplified through PCR to create the DNA library. Quality assurance was conducted through agarose gel electrophoresis before sequencing the library, which was performed using the HiSeq X Ten platform at GENOMICS Co. in Taiwan. The sequenced data were aligned to the reference sequence of *L.* aegyptiaca (ASM1713956v1) using the "mem" command in the BWA software. Subsequent analyses were carried out using GATK software (McKenna et al., 2010) [62]. To ensure the quality of SNP discovery, SNPs were filtered based on quality score > 20, a missing rate < 0.05, and a minor allele frequency > 0.05. In total, 8,919 SNPs were obtained after applying these criteria.

### Population structure analysis

For population structure analysis, we utilized Structure 2.3.4 software [63]. The burn-in time and Markov Chain Monte Carlo (MCMC) parameters were both set to 1,000 and 10,000, respectively. We explored a range of K values from 2 to 8 and conducted three calculations to assess population clusters. To determine the optimal number of clusters (K), we analyzed the output results using Structure Harvester and calculated Delta K. In this analysis, we employed a total of 8,919 SNPs.

### PCA analysis and GWAS study

A total of 8,919 filtered SNPs were utilized to conduct Principal Component Analysis (PCA) and Genome-Wide Association Study (GWAS). PCA was performed using the R program platform [64], and the first five principal components were chosen as covariates for the subsequent GWAS analysis. The GWAS was carried out using the general linear model (GLM) in TASSEL 5 [65] to investigate potential genetic associations with the target traits. To effectively visualize and interpret the results, we employed the qqman package [66] in R to generate Manhattan plots and boxplots, enabling us to explore the distribution and significance of genetic variants across the genome.

### Development and validation of AUDPC-associated SNP marker

An AUDPC-associated SNP was identified, and a SNP marker was developed based on the flanking region sequence. If the primer's end can complement the SNP, PCR amplification can be employed to confirm the presence of the SNP type at that specific site. To validate the accuracy of this AUDPC-associated SNP marker, another population consisting of 21 luffa individuals from *L. aegyptiaca* Mill was selected. Expected genotypes were estimated based on the phenotypes observed 21 days after Fusarium inoculation, and observed genotypes were obtained through SNP detection using PCR. The accuracy of the marker was assessed by calculating the ratio of observed genotypes with specific phenotypes to expected genotypes with specific phenotypes.

### Supplementary Information

**Supplementary Material 1.**

## Data Availability

All datasets used and/or analyzed during the current study are available in the NCBI database with accession number PRJNA1009245.

## Abbreviations

GATK: Genome analysis toolkit
GLM: General linear model
GWAS: Genome-wide association study
MCMC: Markov Chain Monte Carlo
PCA: Principal component analysis
qPCR: Quantitative real-time reverse transcription-polymerase chain reaction
sp-GWAS: Single plant GWAS
