# Supplementary material for: Analyzing genetic diversity in luffa and developing a Fusarium wilt-susceptible linked SNP marker through a single plant genome-wide association (sp-GWAS) study
Source: BMC Plant Biol. 2024 Apr 22;24:307. doi: 10.1186/s12870-024-05022-7 (PMC11034075; doi:10.1186/s12870-024-05022-7)
Supplement: Supplementary file 1 — Supplementary Material 1. [file 12870_2024_5022_MOESM1_ESM.pdf]

Table S1 The phenotypes of 97 collected luffa individuals

| Sample | Species | Country | Hypocotyl l | Hypocotyl w | Plant height | Stem width | AUDPC | R/S |
|---|---|---|---|---|---|---|---|---|
| 1 | I | Philippines | 95.24 | 2.51 | 537 | 1.77 | 87.5 | S |
| 2 | I | Philippines | 85.79 | 2.34 | 142 | 1.4 | 31.5 | S |
| 3 | I | Philippines | 98.28 | 2.47 | 453 | 1.59 | 31.5 | S |
| 4 | I | Philippines | 88.72 | 2.45 | 237 | 1.54 | 31.5 | S |
| 5 | II | Philippines | 69.71 | 2.09 | 453 | 1.2 | 59.5 | S |
| 6 | II | Philippines | 85.12 | 3 | 690 | 1.92 | 10.5 | S |
| 7 | II | Indonesia | 64.02 | 2.76 | 69 | 2.47 | 59.5 | S |
| 8 | II | Indonesia | 71.82 | 2.54 | 430 | 2.47 | 59.5 | S |
| 9 | II | Indonesia | 72.31 | 2.98 | 370 | 2.03 | 17.5 | S |
| 10 | II | Indonesia | 84.69 | 2.89 | 1135 | 2.48 | 87.5 | S |
| 11 | I | Vietnam | 104.45 | 2.59 | 663 | 1.68 | 87.5 | S |
| 12 | II | USA | 114.1 | 3.22 | 453 | 2.07 | 66.5 | S |
| 13 | I | Vietnam | 100.31 | 2.59 | 633 | 1.8 | 59.5 | S |
| 14 | I | Vietnam | 109.73 | 2.72 | 1052 | 2.46 | 87.5 | S |
| 15 | I | Vietnam | 85.02 | 2.75 | 142 | 1.79 | 87.5 | S |
| 16 | I | Vietnam | 106.34 | 1.83 | 563 | 1.54 | 87.5 | S |
| 17 | I | Vietnam | 87.62 | 2.56 | 543 | 1.78 | 59.5 | S |
| 18 | I | Laos | 81.61 | 2.08 | 651 | 1.5 | 59.5 | S |
| 19 | I | Laos | 79.05 | 3.03 | 1000 | 1.39 | 59.5 | S |
| 20 | I | Laos | 83.03 | 2.38 | 431 | 1.54 | 59.5 | S |
| 21 | I | Vietnam | 81.33 | 2.27 | 520 | 2.41 | 87.5 | S |
| 22 | I | Vietnam | 114.88 | 2.39 | 382 | 1.51 | 87.5 | S |
| 23 | I | Vietnam | 79.8 | 2.89 | 648 | 1.78 | 59.5 | S |
| 24 | I | Bangladesh | 51.87 | 2.3 | 563 | 1.77 | 59.5 | S |
| 25 | I | Bangladesh | 60.81 | 2.69 | 233 | 2.03 | 59.5 | S |
| 26 | I | Bangladesh | 69.68 | 2.19 | 314 | 1.54 | 59.5 | S |
| 27 | II | Taiwan | 105.09 | 2.76 | 1000 | 2.25 | 59.5 | S |
| 28 | II | Taiwan | 118.17 | 2.56 | 491 | 1.94 | 28 | S |
| 29 | II | Taiwan | 97.13 | 2.62 | 690 | 1.72 | 59.5 | S |
| 30 | I | Taiwan | 88 | 2.46 | 852 | 1.63 | 0 | R |
| 31 | I | Taiwan | 72.77 | 2.22 | 314 | 2.25 | 59.5 | S |
| 32 | I | Taiwan | 81.84 | 2.25 | 651 | 1.54 | 0 | R |
| 33 | I | Laos | 65 | 2.3 | 197 | 1.81 | 24.5 | S |
| 34 | I | Laos | 90.52 | 2.35 | 293 | 2.03 | 59.5 | S |
| 35 | I | Laos | 91.71 | 2.28 | 549 | 1.48 | 59.5 | S |
| 36 | I | Laos | 82.91 | 2.74 | 633 | 1.52 | 17.5 | S |
| 37 | I | Laos | 89.95 | 2.73 | 142 | 2.16 | 59.5 | S |
| 38 | I | Laos | 72.15 | 2.9 | 197 | 2.4 | 59.5 | S |
| 39 | I | Laos | 73.82 | 2.46 | 237 | 1.54 | 59.5 | S |
| 40 | I | Laos | 57.05 | 2.55 | 453 | 1.38 | 59.5 | S |
| 41 | I | Laos | 70.44 | 2.36 | 237 | 2.03 | 59.5 | S |
| 42 | I | Uzbekistan | 92.57 | 1.84 | 690 | 1.29 | 59.5 | S |
| 43 | I | Cambodia | 66.69 | 2.53 | 453 | 1.25 | 66.5 | S |
| 44 | II | Malaysia | 78.03 | 2.69 | 1052 | 2.4 | 0 | R |
| 45 | II | Malaysia | 90.07 | 3.78 | 650 | 2.23 | 0 | R |
| 46 | II | Malaysia | 65.6 | 3.45 | 453 | 2.41 | 17.5 | S |
| 47 | I | Malaysia | 111.15 | 2.89 | 1135 | 2.28 | 0 | R |
| 48 | II | Malaysia | 80.42 | 3.56 | 453 | 1.2 | 38.5 | S |
| 49 | II | Malaysia | 74.83 | 3.81 | 735 | 1.77 | 17.5 | S |

| | | | | | | | | |
|---|---|---|---|---|---|---|---|---|
| 50 | II | Malaysia | 79.69 | 2.61 | 648 | 1.64 | 59.5 | S |
| 51 | II | Malaysia | 54.05 | 3.4 | 680 | 1.79 | 17.5 | S |
| 52 | I | Malaysia | 57.01 | 2.67 | 542 | 1.79 | 0 | R |
| 53 | I | Philippines | 37.81 | 2.74 | 497 | 1.82 | 17.5 | S |
| 54 | II | Philippines | 58.73 | 3.71 | 690 | 1.78 | 17.5 | S |
| 55 | I | Philippines | 62.11 | 2.18 | 69 | 1.54 | 66.5 | S |
| 56 | I | Philippines | 59.25 | 2.46 | 651 | 1.68 | 66.5 | S |
| 57 | I | Philippines | 64.7 | 2.4 | 710 | 1.16 | 17.5 | S |
| 58 | I | Philippines | 61.1 | 2.57 | 537 | 1.28 | 38.5 | S |
| 59 | I | Philippines | 67.16 | 2.51 | 537 | 1.79 | 87.5 | S |
| 60 | I | Philippines | 81.32 | 2.43 | 732 | 1.24 | 10.5 | S |
| 61 | I | Philippines | 49.21 | 2.36 | 663 | 1.45 | 66.5 | S |
| 62 | I | Philippines | 57.11 | 2.41 | 665 | 1.48 | 73.5 | S |
| 63 | I | Philippines | 90.25 | 2.37 | 643 | 1.5 | 59.5 | S |
| 64 | II | Philippines | 71.65 | 2.69 | 915 | 2.13 | 17.5 | S |
| 65 | I | Philippines | 81.91 | 2.22 | 69 | 1.74 | 31.5 | S |
| 66 | I | Philippines | 40.95 | 2.25 | 314 | 1.81 | 66.5 | S |
| 67 | I | Philippines | 66.83 | 2.27 | 796 | 1.77 | 0 | R |
| 68 | I | Philippines | 72.72 | 2.17 | 510 | 1.2 | 59.5 | S |
| 69 | I | Philippines | 52.79 | 2.14 | 539 | 1.73 | 0 | R |
| 70 | II | Philippines | 60.78 | 2.69 | 482 | 1.49 | 17.5 | S |
| 71 | I | Philippines | 76.25 | 2.94 | 520 | 1.94 | 66.5 | S |
| 72 | I | Philippines | 78.78 | 2.43 | 488 | 1.2 | 45.5 | S |
| 73 | I | Philippines | 67.01 | 2.41 | 682 | 1.45 | 0 | R |
| 74 | I | Philippines | 33.27 | 2.42 | 690 | 1.92 | 38.5 | S |
| 75 | I | Philippines | 73.54 | 2.28 | 282 | 1.89 | 0 | R |
| 76 | III | Philippines | 97.27 | 2.89 | 1100 | 2.07 | 45.5 | S |
| 77 | I | Philippines | 83.15 | 3.02 | 648 | 1.21 | 87.5 | S |
| 78 | II | Thailand | 84.82 | 2.86 | 142 | 1.79 | 45.5 | S |
| 79 | II | Philippines | 114.7 | 2.92 | 676 | 1.78 | 0 | R |
| 80 | I | Philippines | 89.79 | 2.14 | 69 | 1.71 | 38.5 | S |
| 81 | I | Philippines | 112.52 | 2.68 | 431 | 1.58 | 17.5 | S |
| 82 | II | Thailand | 100.72 | 3.22 | 563 | 1.54 | 52.5 | S |
| 83 | II | Thailand | 73.64 | 3.25 | 452 | 1.48 | 0 | R |
| 84 | II | Thailand | 65.41 | 3.04 | 142 | 1.92 | 66.5 | S |
| 85 | II | Thailand | 108.54 | 2.81 | 543 | 1.78 | 59.5 | S |
| 86 | II | Thailand | 102.25 | 3.22 | 491 | 1.49 | 66.5 | S |
| 87 | I | Vietnam | 95.6 | 3.08 | 603 | 0.86 | 0 | R |
| 88 | II | Thailand | 122.46 | 2.69 | 382 | 1.2 | 10.5 | S |
| 89 | II | Thailand | 77.3 | 2.46 | 852 | 1.4 | 59.5 | S |
| 90 | II | Thailand | 109.74 | 2.5 | 1064 | 1.96 | 0 | R |
| 91 | II | Thailand | 122.56 | 2.91 | 507 | 1.25 | 17.5 | S |
| 92 | II | Thailand | 106.82 | 3.5 | 1364 | 2.4 | 59.5 | S |
| 93 | I | Thailand | 74.62 | 2.64 | 237 | 1.89 | 59.5 | S |
| 94 | I | Taiwan | 42.63 | 2.85 | 233 | 1.71 | 66.5 | S |
| 95 | II | Taiwan | 82.14 | 2.68 | 735 | 1.54 | 66.5 | S |
| 96 | I | Taiwan | 66.87 | 2.57 | 453 | 1.58 | 66.5 | S |
| 97 | I | Taiwan | 68.58 | 2.38 | 150 | 1.81 | 38.5 | S |

I: Indicates species *Luffa_aegyptiaca* Mill.
II: Indicates species *Luffa_acutangula_(L.)*ROXB.
III: Indicates species *Luffa*_spp.

Hypocotyl l: Indicates hypocotyl length
Hypocotyl w: Indicates hypocotyl width
R/S: Resistant or Susceptible phenotype of luffa individual on 21 days after inoculation

Table S2 The primer sequence designed from AUDPC-associated SNPs

| SNP ID | Chromosome and position | SNP | Type | Forward 5' | Reverse 5' |
|---|---|---|---|---|---|
| S2_11917148 | 2:11917148 | T/G | Wildtype | AGTCATTGTGTTTTTCCTGTTTCTT | GCATCAATCACTTCAAATCCCCTC |
| | | | Mutant | AGTCATTGTGTTTTTCCTGTTTCTG | GCATCAATCACTTCAAATCCCCTC |
